# New insights explain that organic agriculture as sustainable agriculture enhances the sustainable development of medicinal plants

**DOI:** 10.3389/fpls.2022.959810

**Published:** 2022-09-30

**Authors:** Linlin Jiang, Yuan Chen, Xiaoqin Wang, Wenfang Guo, Yaqiong Bi, Chunhong Zhang, Jianhua Wang, Minhui Li

**Affiliations:** ^1^Inner Mongolia Autonomous Region Hospital of Traditional Chinese Medicine, Inner Mongolia Medical University of Clinical College of Traditional Chinese Medicine, Hohhot, China; ^2^Department of Pharmacy, Inner Mongolia Medical University, Hohhot, China; ^3^Inner Mongolia Institute of Traditional Chinese and Mongolian Medicine, Hohhot, China; ^4^Department of Pharmacy, Baotou Medical College, Baotou, China; ^5^Inner Mongolia Key Laboratory of Characteristic Geoherbs Resources Protection and Utilization, Baotou, China

**Keywords:** medicinal plants, organic agriculture, sustainable development, systematic assessment, environmental improvement

## Abstract

As global health care demand continues to increase, medicinal plant productivity must progress without exhausting critical environmental resources. Hence, it is important to explore practices that can improve the quality, safety, and sustainability of medicinal plants, as well as ecological stability. Organic farming has recently gained significance as a sustainable cultivation alternative owing to increased awareness of the adverse effects of conventional cultivation method. Here, this study aimed to investigate the feasibility of organic farming as a solution for sustainable cultivation of medicinal plants from multiple perspectives and long-term benefits to the environment. Organic agricultural practices of medicinal plants were evaluated from a multi-dimensional perspective (environment, economy, and society) using extensive research data and literature and field surveys. Data from medicinal plant cultivation in Inner Mongolia were acquired for 76 sites from four data stations between 2014 and 2021. Data analysis revealed that organic medicinal plants can improve safety by reducing pesticide exposure risks. Simultaneously, organic agriculture of medicinal plants can improve biodiversity by effectively reducing pesticide and fertilizer use, which also provides natural safe products for health care. With the improvement of quality, the retail price will have a certain advantage, which will improve the income of farmers. Moreover, organic agriculture enhanced profitability because of the higher organic premium on medicinal plant products and improved ecosystem stability by increasing plant diversity. The findings of this study suggest that organic cultivation strategies can improve the quality and safety of medicinal plants and further provide a basis for promoting the sustainable development and ecological stability of medicinal plants. However, not all medicinal plant cultivators are guaranteed to adopt organic farming practices, but if all technological elements are correctly applied, the system can be maintained sustainably to expand the area of organically cultivated plants in the future.

## Introduction

Therapeutic properties of medicinal plants are well-known and are the basis of modern medicines. Currently, nearly 85% of the world’s population relies on medicinal plants as their primary source of health care (Adhikari et al., 2021). Furthermore, medicinal plants are also frequently used as dietary supplements, which greatly impact human health (Yao et al., 2018; Chen et al., 2021). They are extensively consumed as foods due to their bioactive constituents and health benefits (Jiang et al., 2021). World Health Organization has estimated that the global trade of medicinal plants and their derivatives will increase to $50 trillion by 2050 (Chen et al., 2021). This is anticipated to cause a rapid increase in global demand for medicinal plants, which can exacerbate the unsustainable utilization of medicinal plants from wild sources. Medicinal plants could play a key role in the balance and stability of the ecosystem (Wang, 2020). Therefore, unsustainable cultivation of medicinal plants will result in environmental and even social problems.

Careful management and artificial cultivation can help create sustainable opportunities for the medicinal plant market (Hinsley et al., 2019; Bi et al., 2020). The current cultivation systems of medicinal plants successfully increase yields but are often associated with an intensive application of massive chemical inputs and tillage (Amani Machiani et al., 2021). These cultivation practices can cause soil degradation, increase the risk of pests and disease outbreaks, reduce biodiversity, and cause other environmental problems (Li et al., 2021). Moreover, the likely uptake of pesticide residues and heavy metals from the soil by the medicinal plants can eventually endanger the safety of consumers (He et al., 2021). In the long term, soil deterioration can also decrease the yield and ultimately affect the agricultural economy (Cui et al., 2021). Although health care demand is increasing, environmental and sustainability issues and the dependence on the existing agricultural model, which uses massive chemical inputs, are major concerns for human communities today (Henneron et al., 2015). Therefore, modern agriculture now faces the challenge of developing sustainable systems that pursue production intensification while greatly mitigating their environmental impacts. This “push” warrants the exploration of a system with enhanced quality and safety for the sustainability of medicinal plants. This will also further improve the ecological environment. In recent years, the advantages of organic agriculture have been widely explored, and despite its lower yields, this cultivation mode is considered a feasible alternative to conventional methods (Connor and Mínguez, 2012; Katayama et al., 2019). Therefore, organic agriculture is frequently advocated as a solution to achieving sustainable plant cultivation (Mie et al., 2017).

Medicinal plants possess several specific characteristics. Because of their unique medicinal properties, the influence of organic cultivation on metabolites should be considered. In this context, significant amounts of agrochemical inputs might reduce the accumulation of beneficial compounds, thus, organic cultivation offers a means to improve the quality of medicinal plants (Guo et al., 2021; Kang et al., 2021). Moreover, natural environments conforming to the native habitat of medicinal plants are ideal for their cultivation. Numerous studies have demonstrated that organic farming systems could potentially restore more favorable conditions than conventional farming systems (Mie et al., 2017), however, reports on their beneficial effects on medicinal plants are scarce. Moreover, studies comparing the favorable effects of organic and conservative versus conventional farming systems on medicinal plants from multiple angles are lacking.

Driven by the 2030 Sustainable Development Goals, here, this study systematically assessed the multi-dimensional impacts of organic agriculture on medicinal plants. This study combined a literature survey with interviews coupled with long-term field investigations and aimed to develop a reference for promoting sustainable organic cultivation of medicinal plants by evaluating the data on the influencing factors and their effects, combined with field and social survey to explore the perspectives of biodiversity, economy, and society. The analysis of different cultivation methods indicated that organic agriculture increases biodiversity and economic benefits and reduces health risks. We hypothesized that organic farming of medicinal plants will yield natural safe products for health care. With appropriate utilization of knowledge and technology, organic cultivation of medicinal plants can be a sustainable and safe alternative to conventional systems.

## Materials and methods

### Experimental design

The literature survey results were used to initially capture the main influencing factors and effects of organic agriculture. In the later stage, literature and field surveys were carried out simultaneously to provide a reference for the sustainable development of the organic cultivation of medicinal plants. The key factors determined from these research methods are listed in Table 1.

**TABLE 1:** Research methods and major themes covered.

Dimensions		Domains of inquiry
Organic agriculture from original articles: identification and selection of indicators		(1) Analysis of the countries on organic agriculture related articles. (2) Visual analysis of the factors, results and final classification of organic and conventional agriculture (3) Established the decision tree model of influencing factors and results
Fieldwork research	Economy	Planting area, output, retail price, output value input cost (including seedling, land rent, chemical fertilizers, pesticides, herbicides and other inputs, labor costs), output income (including output value, yield), net income, input-output ratio,
	Environment	Pests diseases, grass damage, the use of chemical fertilizers, pesticides and herbicides, species richness (biodiversity),
	Society	The number of pesticide poisoning.

#### Organic agriculture from original articles: Identification and selection of indicators

To determine the characteristics and impacts of organic agriculture, this study collected all research papers from a search of the Web of Science Core Collection and used the search terms: “organic agriculture,” “conventional agriculture,” or “traditional agriculture,” from January 2000 to December 2021. Articles containing practices, outcomes, or both were selected. In total, 601 papers of interest were identified. Furthermore, to determine the correlation between agricultural practices and outcomes, 128 articles were selected for an in-depth analysis (Supplementary Table 1). Based on the common terms and topics covered, this study classified the articles into three main subject areas: (i) environment, (ii) economy, and (iii) society. Based on the Web of Science database, the “CiteSpace” (Chen, 2004) analysis tool was used to cluster the “organic agriculture” related articles country-wise. The freely available Java application of CiteSpace (version 5.8.R3) was used to comprehensively analyze the source countries of the research publications. Then, the Sankey diagram was used to conduct a visual analysis of the factors, resultant effects, and final classification of organic and conventional agriculture and determine the relevant indicators affecting organic and conventional agriculture. This analysis highlighted the main impact indicators of the gap between organic and conventional agriculture of medicinal plants and identified planting patterns to improve the sustainable and safe cultivation of medicinal plants. Furthermore, a decision tree model was established to analyze the influencing factors and results of organic and conventional agriculture.

#### Fieldwork research

Inner Mongolia in North China extends diagonally from northeast to southwest in a long, narrow shape. It stretches 2,400 km from east to west and covers an area of 1.183 million km^2^. Inner Mongolia is an important planting region of medicinal plants because the medicinal plant resources in the northeastern parts are moisture-loving and cold-resistant, while those in the southwest are light-loving, drought-tolerant, and adaptable. Therefore, this region was selected for the study area.

**FIGURE 1:**
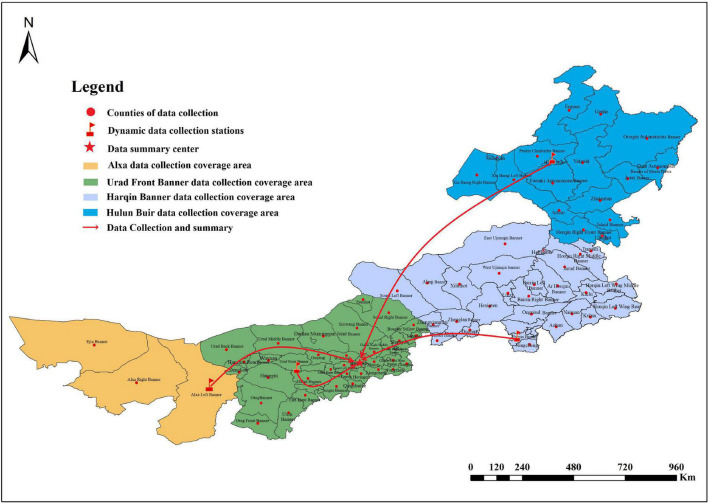
The framework for data collection methods.

The cultivation of medicinal plants in Inner Mongolia has been investigated since September 2014. The study involved both field- and interview-based investigations. Based on county-level administrative units, data was collected, and the information was analyzed in accordance with the method of “one data summary center + four dynamic data collection stations.” Data from 76 surrounding counties was first collected by four dynamic data collection stations (including Alxa Left Banner, Urad Front Banner, Harqin Banner, and Hulun Buir) and then submitted to the data collection center (Hohhot). Next, the data were analyzed (Figure 1), and the survey results of the medicinal plants were summarized by the data collection center.

Data were collected from September 2014 to December 2021 through random semi-structured and structured interviews and stakeholders in the study region. The investigation was based on data collected for the medicinal plants output in the planting region, including retail price, and value to acquire a comprehensive and systematic understanding of the socio-economic impacts of quality and cost of the local medicinal plant resources. The input (including seedlings, land rent, chemical fertilizers, pesticides, herbicides, and labor costs) and output costs (including output value and yield), net income, and input-output ratio of organic and conventional planting were systematically recorded. Simultaneously, data for the number of pesticide poisoning cases were collected from local hospitals. The field investigation was based on an in-depth analysis of the responses to interview questions aimed at determining the cultivation characteristics of medicinal plants. During this period, several representative medicinal plants (and their respective organic planting patterns) were selected for analysis, including *Paeonia lactiflora* Pall., *Saposhnikovia divaricata* (Turcz.) Schischk, *Cistanche deserticola* Ma., *Astragalus mongholicus* Bunge, and *Glycyrrhiza uralensis* Fisch., and long-term field observation experiments were conducted on them. The growth environment, species richness (biodiversity), pests and diseases, grass damage, field management, and yield of these five medicinal plants were monitored and recorded for organic and traditional cultivation systems. Simultaneously, this study also conducted research on seed and seedling breeding and standard planting system to obtain basic information for policy development for the sustainable development of medicinal plant resources.

Furthermore, we selected 10 m × 10 m quadrats for the analysis of plant biodiversity. In this research, we measured biodiversity by species richness (number of species of all plants contained in an area of *A. mongholicu* or *G. uralensis*). Moreover, the data of medicinal plant quadrats collected by the field survey were analyzed, and the numbers of medicinal plant species under traditional and organic cultivation modes were compared. We also investigated the use of chemical fertilizers, pesticides, and herbicides under these two modes and the key factors influencing their mechanisms and effectiveness. The correlation between biodiversity and organic planting was discussed, which provided reference for the stability of the ecological environment.

### Environmental quantification research and analysis

The indicators of farming performance and environmental impacts (differences between organic and conventional agriculture per hectare of farmland area) were obtained from the literature (Meng et al., 2017). This research mainly included *P. lactiflora* (1,333 ha), *C. deserticola* (3,333 ha), and *A. mongholicus* (1,333 ha). To obtain the economic value associated with farming performance and environmental impacts of organic and conventional cultivation of medicinal plants, the area of organic arable land was first multiplied by the price of each performance or impact indicator. This study presented several sources of uncertainty; for example, there are few studies on medicinal plants worldwide; thus, the reported data varied greatly according to several specific factors. For each indicator, this study conducted a literature survey, identified the range of indicator values, and set an appropriate value within the medicinal plant agricultural context.

### Questionnaire research and analysis

A structured questionnaire was formulated based on a literature survey involving perception, attitude, behavior, and intention of organic food and consumption, as well as other studies associated with research in ecological planting. This questionnaire is part of more significant research aimed at investigating the attitude and cognition of ecological planting from the perspective of different people. The survey was conducted with a random sampling approach in August 2019. Questionnaires also enable researchers to establish, distribute, collect, and manage their data online. Our questionnaire consisted of four parts: (i) demographic profiles of the respondents; (ii) health/risk perceptions and attitudes toward a traditional planting pattern; (iii) the consciousness and attitudes toward an organic planting pattern; and (iv) perceived change in future planting patterns: stability of attitudes toward an ecological planting pattern. Supplementary Table 2 shows the socio-demographic profiles of the respondents. A total of 1,006 usable responses were collected for data analysis.

### Statistical analyses

#### Analytic hierarchy process

Analytic hierarchy process (AHP) is the decomposition of decision-related elements into goal, criteria, and scheme levels (Zhang et al., 2016a). AHP analyzes the influencing factors of each level and calculates the weight—the greater the factor weight, the greater its influence on the problem. AHP is a systematic analysis method, and the influence degree of each factor at each level on the result is clear and quantified (Zhao et al., 2021); it can organically link qualitative and quantitative methods, decompose complex systems, and focus more on qualitative analysis and discrimination than ordinary quantitative methods (Aykut, 2021). In this study, we qualitatively and quantitatively analyzed the economic benefits of traditional and organic cultivation modes for medicinal plants using AHP. Cultivation mode was considered as the target, and input and output were considered the criterion layers and named A1 and A2, respectively. AHP was applied in four major steps. (1) Appropriate weights were assigned to the parameters according to interview survey and literature reviews. (2) A pairwise comparison between the parameters was developed, and the scale for pairwise comparison developed by Saaty was used to compare the parameters (Aykut, 2021). The relative importance of parameters on the benefits of cultivation modes were calculated based on a nine-point hierarchy scale (Zhao et al., 2021). (3) Parameter weights were calculated using the arithmetic average method. The consistency of judgment matrix was normalized; thus, the index weight vector could be calculated. The maximum eigenvalue λ_*max*_ of the decision matrix was obtained using Equation 1:


$$\lambda \,\text{max}=\frac{1}{n}\,\sum_{i=1}^{n}\frac{\left(A\,\omega \right)\,i}{W\,\text{i}}$$

After calculating the weight, a consistency test for this factor was conducted. The consistency test index is CI, which is calculated using Equation 2:


CI=(λ-m⁢a⁢xn)*(n-1)

(4) A consistency ratio (CR) was calculated according to RI and CI to judge the rationality of weight allocation. A CR < 0.1 is considered a consistent principle of consistency. The CR is calculated using Equation 3:


C⁢R=C⁢I/R⁢I.

#### Two-level logistic regression

The two-level logistic regression model is an important method to study the dependent variables as dichotomous variables and is a suitable regression analysis method for qualitative variables. The two-level logistic regression model corresponding to the independent variables was described earlier (Talukder and Hossain, 2020) and is present in Equation 4:


$$P\left(Y=1\right)=\frac{\exp\left(\beta \,0+\beta \,1\,X\,1+\beta \,2\,X\,2+\ldots \,\beta \,n\,X\,n\right)}{1+\exp\left(\beta \,0+\beta \,1\,X\,1+\beta \,2\,X\,2+\ldots \,\beta \,n\,X\,\text{n}\right)}$$

where β_0_ is a constant term (or intercept), X_*i*_ represents each influencing factor, and β_*i*_ is the partial regression coefficient corresponding to X_*i*_. Furthermore, under the condition that other independent variables are constant, the independent variable X_*n*_ changes by a unit, and the dependent variable corresponding to the odds ratio averages in exp units. Logarithmic transformation of the odds ratio to obtain a linear model of the two-level logistic regression model is calculated using Equation 5 (Park and Lake, 2005):


$$\ln\frac{p\,i}{1-p\,i}=\text{logit}\,P=\beta \,0+\beta \,1\,X\,1+\beta \,2\,X\,2+\ldots \,\beta \,n\,X\,\text{n}$$

The two-level logistic regression model is mainly used in regression analysis where the dependent variable is a categorical variable, and the independent variables that impact the dependent variable can be selected from multiple independent variables. In this study, organic farming was selected as the dependent variable. SPSS statistical software (version 20.0) was used to conduct the two-level logistic regression analysis on the survey data of the respondents in the questionnaire. Age, gender, education level, occupation, and family income were selected as individual characteristic factor variables. Simultaneously, this study selected three additional variables, health/risk attitudes (the satisfaction degree of traditional production), cognition and attitude (the sense of trust), and commodity attribute (acceptable price of organic cultivation mode production) for future reference. It should be noted that as the main producers, farmers are the final executor of the organic agricultural system with medicinal plants. Therefore, this study tried to consider the six variables (pesticides, fertilizers, herbicides, biodiversity, input/output ratio, and pesticide poisoning) mentioned above to determine the impact of organic agriculture on producers, to provide further evidence for organic cultivation of medicinal plants.

#### Joinpoint regression program

The joinpoint regression program describes changes in data trends by connecting several different line segments on a log-scale at joinpoints. Analysis starts with the minimum number of joinpoints and tests for model fit with a maximum number. In addition, an annual percent change (APC) for each line segment and the corresponding 95% confidence interval were estimated. In the final model, each joinpoint informs a statistically significant change in trends (increases or decreases). To analyze the temporal trends and to colligate significant changes in the organic agricultural land and organic share of the agricultural land, this study performed a joinpoint regression analysis using the joinpoint regression program (Al Hasan et al., 2020). Using this program, we were able to identify the years when a significant change in the linear slope of the trends in the organic agricultural land and organic share of the agricultural land was detected over the study period, from 1999 to 2019. These data are from the FiBL-IFOAM-SOEL surveys (Supplementary Table 3). The joinpoints, were selected when the rate changed significantly (*P* < 0.05). In each joinpoint, the trend significantly alters its direction and observes the transformation or changes in the organic agricultural land and organic share of the agricultural land. Hence, each joinpoint acts as a point of change in the organic agricultural land and organic share of the agricultural land and is the basis of the transformation observed. The analysis began with the minimum number of joinpoints and tested whether one or more joinpoints were statistically significant and must be added to the model. To set forth the linear trends by period, we then calculated the estimated APC for each of those trends in the organic agricultural land and organic share of the agricultural land.

#### Generalized additive models

Generalized additive models (GAM) have been widely used in the non-linear relationship between response variables and multiple explanatory variables. They can be used to analyze interactions between influencing factors on the response variables. GAM allow the relationships between the explanatory (henceforth covariates) and response variables to be described by smooth curves (usually splines, but potentially other structures). In general, these models are generated using Equation 6 (Jiang et al., 2018):


$$\text{g}\,\left(\mu \right)=\beta \,0+\sum_{i=1}^{k}f\,i\,\left(x\,i\right)$$

where g(μ) is a link function, β_0_ is a constant, fi(xi) is a smoothing function that describes the relationship between g(μ) and the *i*-th factor, and *k* is the number of explanatory factors. The Akechi information criterion (AIC) was used to select suitable models with different explanatory variables. We used RStudio (version 2021.09.0) to build and evaluate a GAM. We identified five economic interaction factors that potentially affect the organic share in agriculture using a non-parametric model. We selected representative countries, including the top 30 organic share countries and the top 20 research hot-spots, and there were 40 countries in total. We mainly collected five variables, namely, GDP, GDP per capita, arable land, the added value of the share of agricultural land to total GDP, and industrial added value to total GDP. These data come from FiBL-IFOAM-SOEL and https://www.worldbank.org/en/home (Supplementary Table 4).

## Results

### Organic agriculture characteristics

**FIGURE 2:**
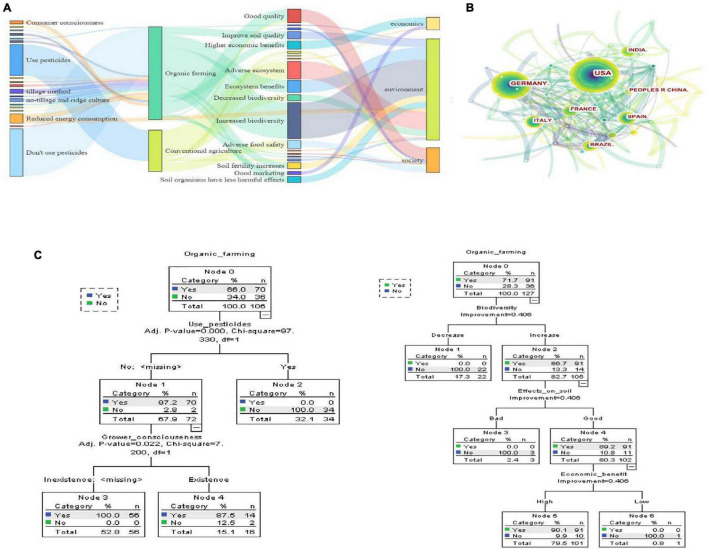
The development trend and research focus of organic agriculture over the past 21 years (Data are provided in the Supplementary Table 1). **(A)** The sankey diagram of the factors, results and final classification of organic and conventional agriculture. **(B)** The countries of organic agriculture-related publications. **(C)** The decision trees of major the influencing factors and results of organic and conventional agriculture.

The literature survey data analysis revealed that approximately half of the studies from January 2000 to December 2021 investigated pesticide usage effects, and more than 70% of the studies were explicitly focused on the environment, whereas only 27% considered social and economic outcomes (Figure 2A). This study also reviewed the trend of research focusing on organic agriculture development over the past 20 years of the twenty first century. Most organic agriculture-related publications were from USA, Germany, and Spain (Figure 2B). Moreover, a decision tree analysis of the main factors and outcomes revealed several potential benefits of organic agriculture, including increased biodiversity and soil quality improvement. Research trends in related fields were associated with environmental factors (Figure 2C). However, only a few studies investigated the organic cultivation of medicinal plants. In this study, we address the key findings and discuss the impacts of organic cultivation of medicinal plants on all three areas: social, economic, and environment.

### Field and social survey on medicinal plants

#### Enhanced biodiversity

**FIGURE 3:**
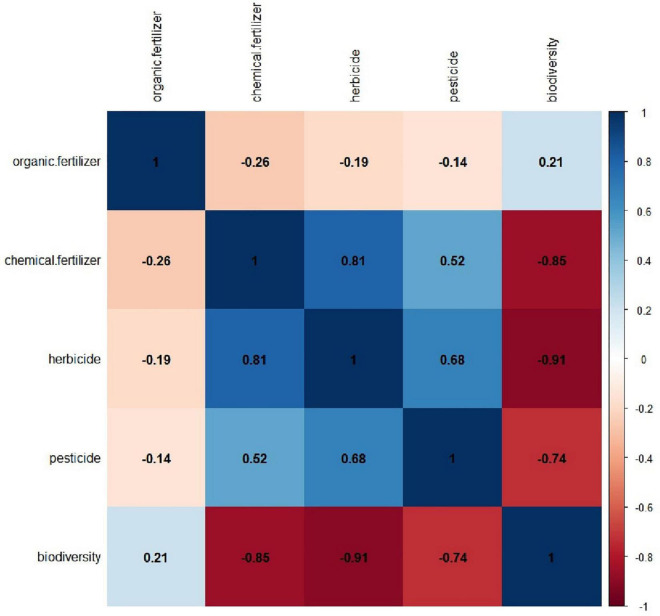
The correlation analysis between agrochemical and biodiversity.

There were 19 plant species in the conventional planting field of *A. mongholicus*, mainly from Chenopodiaceae and Poaceae families, among which *Chenopodium glaucum* L. and *Setaria virid*is (L.) Beauv. were the most abundant. In the organic cultivation fields, 96 other plant species were identified in addition to traditional weeds. The number of plant species in organic fields were more than six times higher than that in the traditional fields. In addition, the results for the *G. uralensis* field also showed higher species richness under organic cultivation (78 species) than under conventional cultivation (15 species). Therefore, organic agriculture can be substantially more advantageous in terms diversity of other plant species in the organic cultivation of medicinal plants. In the correlation analysis, the diversity of medicinal plant fields was significantly negatively correlated with the use of agrochemicals (Figure 3).

#### Increase in economic benefits over time

**FIGURE 4:**
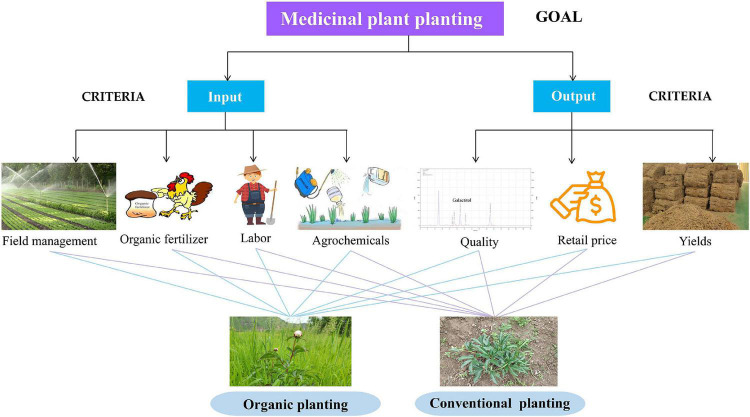
Hierarchical model of the evaluation system of benefits of medicinal plant cultivation.

The hierarchical structure is illustrated in Figure 4. In the combination consistency test, the results were valid for CR < 0.1. All parameters were compared in a pairwise comparison matrix (Supplementary Table 5), which depicts the assigned weightages of the parameters after the calculation and normalization of parameter weightage. The matrix calculation (Supplementary Tables 6, 7) suggests that organic agriculture of medicinal plants is more beneficial than conventional cultivation methods (M_organic agriculture_ = 0.909076; M_conventional agriculture_ = 0.615388).

This study also compared the economic performance of the two cultivation systems of *S. divaricata* (Supplementary Table 8). Organic cultivation of medicinal plants presented a significant decrease in yield, but this was offset by the higher retail prices that resulted in better economic prospects for organic cultivation. At the same time, this study found that when actual organic premiums were applied, gross returns, input/output ratios, and net profit values were significantly higher for *S. divaricata*. Therefore, the organic cultivation of *S. divaricata* has the potential for greater benefits than conventional farming.

#### Greater social sustainability in medicinal plant organic agriculture

##### Social cognitive

At the end of the supply chain, consumer demand can directly or indirectly affect the operations and their interactions throughout the supply chain. According to the characteristics of consumer demand for organic food, this study applied a two-level logistic regression model to analyze the willingness of consumers to support organic agriculture. Among the eight variables, six selected covariates were statistically significant (*P* < 0.05), as shown in Supplementary Table 9. The logistic regression equation was as follows Equation 7:

LogitP = 0.307*(age)+0.523*(education)+0.742*(income/year)-1.279*(health risk attitudes)+2.733*(cognition and attitude)-0.750*(retail price).

The results indicate that the willingness to support organic cultivation of medicinal plants increased with the increasing age of the participants (OR = 1.359, *P* < 0.05). Moreover, a lower perceived health risk led to a higher willingness (*P* < 0.01). Furthermore, respondents 29 years and younger showed a comparatively more positive attitude toward conventional planting patterns than the other age groups. In addition, the willingness was higher for participants with higher education levels (OR = 1.688, *P* < 0.01). Most participants who did not support organic agriculture and considered such changes in planting patterns as unnecessary presented an education level below the junior college degree. The odds of developing the willingness to support organic products were higher for individuals from high-income families (OR = 2.099, *P* < 0.01) than from low-income families. In addition, more than 66% of the participants were willing to pay higher prices for organic planting of medicinal plants because it has greater health benefits compared to its conventional counterpart. Therefore, with the rapid development of social economy, the willingness of consumers to purchase organic products is likely to increase in the future. In addition to the economic factors that influence consumers’ behavior toward organic products, the rapid increase in consumer demand for organic products is driven by a variety of factors, mostly related to food and farm-worker safety.

##### Trends in organic agriculture development

**FIGURE 5:**
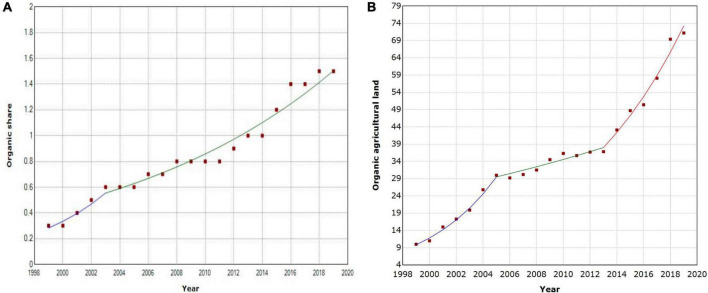
Joinpoint regression analysis of organic share **(A)** and organic agricultural land **(B)** (Source: FiBL-IFOAM-SOEL surveys 1999–2019).

During the 20 years of investigation, the share of organic agricultural land showed a significant (*P* < 0.05) increase at a rapid rate (Figure 5A). Organic agricultural land increased significantly (*P* < 0.05), at 2.9% per year, from 1999 to the early 2005s, and then slowed down with increase at a markedly slow rate (APC = 3.21, *P* < 0.05) until 2013. Subsequently, the trend revived with a marked rise again (APC = 11.55, *P* < 0.05) (Figure 5B). The results revealed a significant increasing pattern over time in the overall trend of organic agricultural land and organic share. Moreover, as the demand for medicinal plants increases, conventional cultivation methods are expected to be more deeply and adversely affected, which should further encourage the development of organic medicinal plant agriculture.

This study found that research related to organic cultivation is heavily biased toward developed countries; therefore, future research in less developed countries should be significantly increased. Considering this background, this study identified five economic interaction factors that can potentially affect the organic share in agriculture using a non-parametric model, GAM, including gross domestic product (GDP), GDP per capita, arable land, the share of agricultural value added to total GDP, and industrial added value to GDP. This study adopted a stepwise method to test the significance of each factor and build the optimal GAM, which is given by the following Equation 8:

(organic_shares)∼ s(GDP) + s(GDP per capita) + s(arable land) + s(agricultural value added).

**FIGURE 6:**
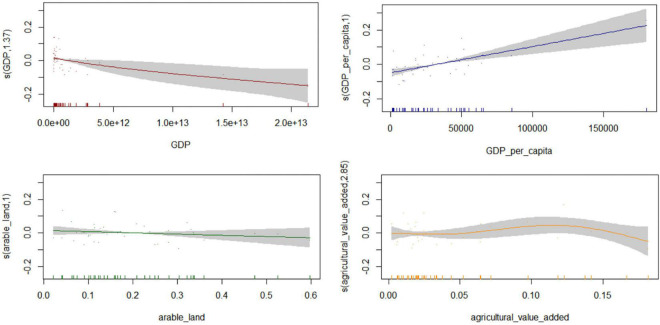
The relationships between driving factors and organic shares.

When the industrial added value to GDP was added, the AIC value increased. Therefore, this study excluded this driving factor when building the GAM. Combined with the model fitting results in Figure 6, arable land and the share of agricultural value added to total GDP had no significant effect on the share of organic agriculture (*P* > 0.05). GDP per capita presented a positive impact on the share of organic agriculture. That is, an increase in these variables is expected to increase the share of organic agriculture (*P* < 0.05).

## Discussion

This study demonstrates that organic agriculture can enhance plant diversity and economic returns for farmers and reduce the risk of pesticide exposure for producers and consumers, which is in line with the global findings from data mining (Seufert and Ramankutty, 2017). This also implies that organic agricultural practices implemented in the cultivation of medicinal plants can improve the ecosystem’s performance to a satisfactory level of social benefits.

In view of global environmental change, a major threat to biodiversity exists due to the loss of habitats and species, which may affect key ecological functions (Vidaller and Dutoit, 2022). Although many field studies have explored crop diversity in organic agriculture, few have directly measured the relationship between organic cultivation and biodiversity associated with medicinal plants (Gomiero et al., 2011; Tamburini et al., 2020). The field studies indicate higher diversity of plants in organic cultivation of medicinal plants, which is consistent with the published data analyzed for organic agricultural crop diversity. Similarly, previous studies have also shown that organic agriculture can promote crop biodiversity through ecological management practices, such as by limiting the use of pesticides (Larsen and Noack, 2021), which was evident in our results. The correlation analysis revealed a strong negative correlation between plant diversity and the use of agrochemicals. This suggests that the use leads to ecosystem damage and further strengthens the idea that organic agriculture can increase plant diversity in medical plant systems and further improve the environment. The positive correlation between biodiversity and ecosystem stability predicted by the insurance hypothesis (García-Palacios et al., 2018) also indicates that the organic cultivation of medicinal plants can contribute to the stability of ecosystems by enhancing biodiversity. In addition, original habitats are more suitable for the growth of medicinal plants because they ensure good quality of products. This highlights the concept that the adoption of organic agricultural practices (e.g., intercropping and imitation of natural conditions) and the prohibition of agrochemicals usage can holistically improve the ecological services of the medicinal plant systems, including maintaining biodiversity and promoting healthy and sustainable development, thereby contributing directly or indirectly to the long-term stability of plant quality (Supplementary Table 10). Furthermore, previous studies have reported that organic agriculture systems consistently lead to better soil quality, less soil erosion, and greater faunal diversity compared with conventional systems (Reganold and Wachter, 2016). However, there are gaps in our research regarding the effects of the organic cultivation of medicinal plants on soil quality and faunal diversity.

The current literature on agronomy innovation focuses mainly on technical changes at the plot scale and fails to consider the institutional plant protection at the regional levels (Schut et al., 2014). The ecological zones have a degree of independence, with enough species and enough range. Furthermore, an ecosystem can regulate itself, creating dynamic equilibrium by interacting with other natural objects (Yu and Cao, 2020). Therefore, studies on reducing the adverse environmental impacts of agriculture, such as herbicide pollution, should be continued within certain ecological domains. In conclusion, organic agriculture of medicinal plants can not only improve the regional environment but also further promote the sustainability of that ecosystem. At the same time, economic and social aspects should be considered along with the environmental benefits to enable coherent and sustainable development, as defined by Emas (2015).

An increase in environmental benefits tends to be coupled with a decrease in productivity. The organic cultivation of medicinal plants is expected to continue to expand globally, primarily because of its overall financial advantage (Tamburini et al., 2020; Amani Machiani et al., 2021; Larsen and Noack, 2021). Numerous studies have compared yield differences between organic and conventional systems, revealing approximately 8–25% lower yields in organic cultivation systems (Reganold and Wachter, 2016; Talukder and Hossain, 2020). The data strengthens the argument that organic farming often produces lower yields than conventional systems. By contrast, there are reports that organically managed farms frequently show higher yields than their conventional counterparts under severe drought conditions because organically farmed soils have a higher water-holding capacity (Reganold and Wachter, 2016). As a result of these abiotic stress properties, organic cultivation will increase the adaptation of medicinal plants to the changing environment, thus increasing the yield and improving the economy. For example, *A. mongholicus* cultivation under wild-simulated conditions led to a 2.5 times higher yield than conventional cultivation (Kang et al., 2018). Therefore, the yield gap between organic and conventional agriculture may be reduced or even eliminated upon the adoption of appropriate agricultural measures because of the simulative habitat cultivation and abiotic stress characteristics of medicinal plants.

Although most organic medicinal plants are associated with lower yields, this effect can be offset by their higher retail prices and potential cost savings from the reduced reliance on non-renewable resources and purchased inputs. Supplementary Table 11 shows the environmental benefits and production performance under the two cultivation modes. The savings in production costs and environmental benefits of organic plants compensated for the 10% economic loss associated with yield reduction. Therefore, the organic cultivation of medicinal plants can potentially lead to greater benefits than conventional farming. Recent studies have demonstrated that organic agriculture can be an economically viable alternative for farmers in current medicinal plant systems. Economic benefits commonly dictate the planting mode chosen by farmers (Zhang et al., 2016b). The field survey indicated that farmers have an incorrect perception that lower yields necessarily lead to lower profits. This influence of conventional crop systems and lack of knowledge about estimating the economic aspects could be major reasons for the lack of organic medicinal plant cultivation, despite their potential for higher profits. Therefore, increasing the dissemination of knowledge to farmers regarding the practical efficiency of organic cultivation can assist in achieving economic sustainability in the organic cultivation of medicinal plants. This includes sharing evidence-based information on the efficiency of practices and cost-benefit analyses, removing potential barriers for farmers, such as up-front costs, access to appropriate equipment, and distribution of products (Tamburini et al., 2020).

Yield is an important sustainability metric, but this issue is complex because the high yields of conventional agricultural systems frequently come at the expense of other sustainability goals, including environmental preservation and public health (Reganold and Wachter, 2016). Pesticide poisoning of farm workers and people handling pesticides results in approximately 300,000 deaths and chronic diseases every year (Ponisio et al., 2015; Reganold and Wachter, 2016; Seufert and Ramankutty, 2017). Data obtained from the hospitals in this study revealed that there were approximately 10 cases of acute poisoning per year caused by agrochemicals in Inner Mongolia. Similar concerns have prompted numerous policy initiatives to promote organic production, such as the “zero growth of pesticides.” Moreover, an important caveat is that conventional farming often increases the exposure of producers and consumers to pesticides and other chemicals. Even if global policies decrease the use of pesticides, these chemicals will likely still be used to obtain yields within the standard limits. Furthermore, pesticides and heavy metals disperse slowly and can remain in the soil and reach the medicinal plants, thereby increasing the associated health risks.

Medicinal plants are believed to possess the ability to cure diseases; thus, their safety and effectiveness cannot be ignored. Concerns about the sustainability and safety of traditional agriculture have encouraged the development of organic medicinal plants. Moreover, the results indicated that GDP per capita presented a positive impact on organic agriculture. However, GDP presented a significantly negative impact on the share of organic agriculture and was also significantly negatively correlated with agricultural value added (*P* < 0.05). The results of the correlation analysis are shown in Supplementary Table 12. Countries with low GDP may be driven by economic and subsistence factors and the need to increase food production to develop the agriculture market, which can directly increase agricultural activities and indirectly increase the respective organic share. However, this study also observed that developed countries allocate more funds to support organic agriculture, so the focus of organic research is still biased toward developed countries. Therefore, there is a need to substantially increase the number of organic cultivation studies in less developed countries (Reganold and Wachter, 2016).

Farmers are the main producers and the final executors of the organic agricultural system of medicinal plants (Wen, 2012). In the past, there has been little to no comprehensive analysis regarding the impact of environmental, economic, and social factors on farmers adopting organic cultivation. In terms of comprehensive benefits, ecological benefits are the premise of organic cultivation, whereas, economic benefits represent its driving force. In addition, social continuity might also lead to social benefits (Kang et al., 2018). Therefore, to further clarify the characteristics of organic cultivation of medicinal plants, this study investigated six variables to determine the impact of organic agriculture on producers. Among the six variables, three selected covariates were statistically significant (*P* < 0.05), as shown in Supplementary Table 13. The logistic regression equation was as follows Equation 9:

LogitP = 0.397*(biodiversity)-6.522*(Input/output ratio)-1.854*(Pesticide poisoning).

**FIGURE 7:**
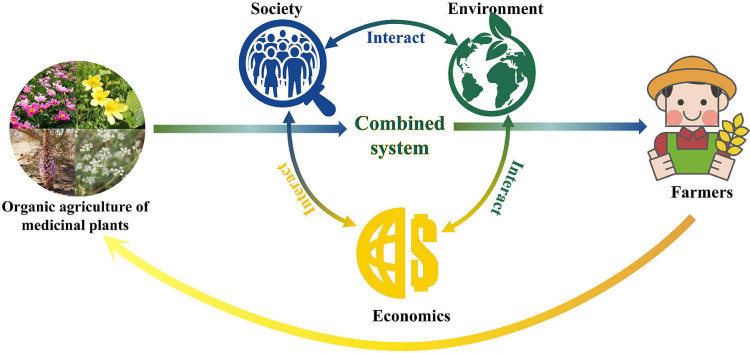
The sustainable development system for organic agriculture of medicinal plants.

The survey indicated that increased biodiversity, increased economic benefits, and reduced health risks of organic agriculture were the main factors driving farmers to select this cultivation mode. This study anticipates that with the development of organic agriculture for medicinal plants, further improvement of biodiversity, economic benefits, and health risks will increase the willingness of farmers to adopt organic cultivation. This would continue to promote the sustainable development of organic agriculture for medicinal plants. For example, the wild-imitating cultivation of *P. lactiflora*, utilizing artificial weeding does not require synthetic chemical products, can significantly increase plant diversity and reduce pesticide exposure, thereby reducing the health risks for farmers and consumers. Furthermore, this cultivation mode can also increase labor input, effectively address the problem of rural surplus labor, and increase farmers’ income. The several aspects of the organic cultivation systems form an interactive model with a clear closed loop. In this comprehensive system, economic, social, and environmental factors complement one another (Figure 7).

## Conclusion

Overall, the data analysis and survey of different cultivation methods indicated that organic agriculture of medicinal plants is an effective method for the sustainable development of medicinal plants. Field surveys revealed that the number of plant species in organic fields were higher than that in traditional fields. By contrast, diversity of medicinal plant fields was significantly negatively correlated with the use of agrochemicals. Therefore, the analysis indicated that organic agriculture of medicinal plants can improve biodiversity by effectively reducing pesticide and fertilizer use, which also provides natural safe products for health care. This study found that although plant yield was reduced under organic cultivation. The gross returns, input/output ratios, and net profit values were significantly higher for *S. divaricata*, considering the actual organic premiums. Organic cultivation of medicinal plants can improve biodiversity and effectively reduce the costs associated with pesticide and fertilizer inputs. Thus, organic cultivation of medicinal plants can also improve long-term returns. At the same time, with the improvement of quality, the retail price will have a certain advantage, which will improve the income of farmers. The most important factor for promoting organic cultivation is the choice of farmers. Our study indicated that increased biodiversity and economic benefits and reduced health risks of organic agriculture were the main factors driving farmers to select this cultivation mode. It also shows that organic cultivation of medicinal plants can be a sustainable and safe alternative to conventional systems.

It should be noted that owing to the specific characteristics of medicinal plants, further research is required to assess whether organic agriculture has the same promoting potential for all the medicinal plants according to their ecological niches. It is also emphasized that medicinal plant systems should be compared cautiously and that the agricultural practices composing the cropping systems of each farming system should be considered in a holistic and comprehensive manner. To achieve this goal, the organic cultivation of medicinal plants must advance while taking advantage of the benefits of the current organic agricultural system and avoiding its drawbacks. Focusing on research pertaining to the specific characteristics of each medicinal plant species will ensure future success. To achieve this goal, further research on organic agriculture-related planting standards and pest and grass control techniques related to organic farming for different medicinal plants is required. Similarly, the advantages of organic cultivation should be illustrated to farmers to motivate them to adhere to the environmental, economic, and social sustainability goals. Furthermore, the area of organically cultivated plants should be increased, but not all medicinal plant farmers are guaranteed to adopt organic farming practices. For the sustainable development of medicinal plants, not only “organic” but also “integrated production system” could be sustainable if all technological elements are properly applied.

## Data availability statement

The original contributions presented in this study are included in the article/Supplementary material, further inquiries can be directed to the corresponding author.

## Author contributions

LJ, YC, and ML conceived the idea and designed the data collection process. YC, YB, CZ, XW, and WG collected and coded the data. LJ, YB, JW, CZ, and ML designed the analysis. LJ, YB, CZ, XW, and YC conducted the analysis. WG, JW, XW, and ML contributed to interpretations of results. All authors wrote the manuscript, read and agreed to the published version of the manuscript, and agreed to be accountable for all aspects of the work in ensuring that questions related to the accuracy or integrity of any part of the work were appropriately investigated and resolved.
