# Supplementary material for: New insights explain that organic agriculture as sustainable agriculture enhances the sustainable development of medicinal plants
Source: Front Plant Sci. 2022 Sep 30;13:959810. doi: 10.3389/fpls.2022.959810 (PMC9562643; doi:10.3389/fpls.2022.959810)
Supplement: Supplementary file 1 [file Data_Sheet_1.doc]

Supplementary Material

Supplementary Table 1 The analysis details of the selected 128 references

| **No.** | **Citation** | **Methods** | | **Measures** | **Rsuluts** | **Types** | | | **Detial** |
| --- | --- | --- | --- | --- | --- | --- | --- | --- | --- |
|  |  | Organic farming | Conventional agriculture |  |  | Environment | Economics | Society |  |
| 1 | Analysis of economic benefits of Chinese medicine eco-agriculture based on multiple stakeholders | Yes | No | Choose organic | Good quality | No | No | Yes | Kang CZ, et al. Zhongguo Zhong Yao Za Zhi. 2021, 46(8): 1858-1863. |
|  |  | Yes | No | Choose organic | Good quality | No | No | Yes |  |
|  |  | Yes | No | Tillage method | Good curative effect | No | No | Yes |  |
| 2 | Effects of Different Land Use Practices on Nitrogen Loss from Runoff During Rainfall Events | Yes | No | Tillage method | Increased biodiversity | Yes | No | No | Luo YF, et al.Huan Jing Ke Xue. 2021, 42(5):2260-2267. |
| 3 | No-tillage:core strategies for sustainable development of ecological agriculture of Chinese materia medica | Yes | No | No-tillage and ridge culture | Increased biodiversity | Yes | No | No | Sun K, et al. Zhongguo Zhong Yao Za Zhi. 2021, 46(8):1869-1875. |
| 4 | Organic agriculture and sustainable development | Yes | No | Choose organic | Soil fertility increases | Yes | No | No | Li Y, Wang G. Ying Yong Sheng Tai Xue Bao. 2004, 15(12):2377-82. |
| 5 | A comparison of the nutritional value and food safety of organically and conventionally produced wheat flours | Yes | No | Choose organic | Good quality | No | No | Yes | Vrček IV, et al. Food Chem. 2014, 143:522-9. |
| 6 | A threshold area ratio of organic to conventional agriculture causes recurrent pathogen outbreaks in organic agriculture | Yes | No | Don't use pesticides | Ecosystem benefits | Yes | No | No | Adl S, Iron D, Kolokolnikov T. Sci Total Environ. 2011, 409(11):2192-7. |
| 7 | Agricultural activities and epidemiology of malaria in Soudano-Sahelian zone in Cameroon | No | Yes | Use pesticides | Health | No | No | Yes | Atangana J, Fomena A, Tamesse JL, Fondjo E. Bull Soc Pathol Exot. 2012, 105(1):23-9. |
| 8 | Agricultural management and labile carbon additions affect soil microbial community structure and interact with carbon and nitrogen cycling | Yes | No | Choose organic | Increased biodiversity | Yes | No | No | Berthrong ST, Buckley DH, Drinkwater LE. Microb Ecol. 2013, 66(1):158-70. |
| 9 | Agricultural management practices influence AMF diversity and community composition with cascading effects on plant productivity | Yes | No | Choose organic | Increased biodiversity | Yes | No | No | Manoharan L, Rosenstock NP, Williams A, Hedlund K. Applied Soil Ecology, 2017, 115: 53-59. |
| 10 | Agricultural practices modulate the bacterial communities, and nitrogen cycling bacterial guild in rhizosphere: field experiment with soybean | Yes | No | No-tillage and ridge culture | Increased biodiversity | Yes | No | No | Singh U, Choudhary AK, Sharma S. J Sci Food Agric. 2021, 101(7):2687-2695. |
|  |  | Yes | No | Use NPK | Increased biodiversity | Yes | No | No |  |
|  |  | Yes | No | Straw retention | Increased biodiversity | Yes | No | No |  |
| 11 | Agroecological strategies for arthropod pest management in Brazil | No | Yes | Use pesticides | Adverse ecosystem | Yes | No | No | Lemos F, Sarmento RA, Teodoro AV, dos Santos GR, do Nascimento IR. Recent Pat Food Nutr Agric. 2011, 3(2):142-54. |
| 12 | An alternative agriculture system is defined by a distinct expression profile of select gene transcripts and proteins | No | Yes | Use pesticides | Increase in production cost | No | Yes | No | Kumar V, Mills DJ, Anderson JD, Mattoo AK. Proc Natl Acad Sci U S A. 2004, 101(29):10535-40. |
| 13 | Ant Communities and Ecosystem Services in Organic Versus Conventional Agriculture in the U.S. Corn Belt | Yes | No | Don't use pesticides | Increased biodiversity | Yes | No | No | Helms JA, Smith J, Clark S, Knupp K, Haddad NM. Environ Entomol. 2021, 23:nvab105. |
| 14 | Antioxidant effectiveness of organically and non-organically grown red oranges in cell culture systems | Yes | No | Choose organic | Good quality | No | No | Yes | Tarozzi A, et al. Eur J Nutr. 2006, 45(3):152-8. |
| 15 | Aquaponics: a sustainable alternative to conventional agriculture in Egypt - a pilot scale investigation | Yes | No | Choose organic | Higher economic benefits | No | Yes | No | El-Essawy H, Nasr P, Sewilam H. Environ Sci Pollut Res Int. 2019, 26(16):15872-15883. |
| 16 | Aqueous and Ethanolic Plant Extracts as Bio-Insecticides-Establishing a Bridge between Raw Scientific Data and Practical Reality | No | Yes | Use pesticides | Adverse ecosystem | Yes | No | No | Tavares WR, Barreto MDC, Seca AML. Plants (Basel). 2021, 10(5):920. |
| 17 | Assessment of mite fauna in different coffee cropping systems in Brazil | Yes | No | Don't use pesticides | Ecosystem benefits | Yes | No | No | Peixoto ML, Fernandes LG, Carvalho MAC, Oliveira MLD, Putti FF, Reis ARD. Biocontrol Science and Technology.2017, 27(3):424-432. |
| 18 | Bamboo Tar as a Novel Fungicide: Its Chemical Components, Laboratory Evaluation, and Field Efficacy Against False Smut and Sheath Blight of Rice and Powdery Mildew and Fusarium Wilt of Cucumber | Yes | No | Biological control agent | Ecosystem benefits | Yes | No | No | Maliang H, Wang P, Chen A, Liu H, Lin H, Ma J. Plant Dis. 2021, 105(2):331-338. |
| 19 | Belowground fungal community diversity, composition and ecological functionality associated with winter wheat in conventional and organic agricultural systems | Yes | No | Don't use pesticides | Increased biodiversity | Yes | No | No | Garnica S, Rosenstein R, Schön ME. PeerJ. 2020, 8:e9732. |
| 20 | Beta diversity at different spatial scales: plant communities in organic and conventional agriculture | Yes | No | Choose organic | Increased biodiversity | Yes | No | No | Gabriel D, Roschewitz I, Tscharntke T, Thies C. Ecol Appl. 2006, 16(5):2011-21. |
| 21 | Biological Control beneath the Feet: A Review of Crop Protection against Insect Root Herbivores | No | Yes | Use pesticides | Adverse ecosystem | Yes | No | No | Kergunteuil A, Bakhtiari M, Formenti L, Xiao Z, Defossez E, Rasmann S. Insects. 2016, 7(4):70. |
| 22 | Cadmium mass balance in French soils under annual crops: Scenarios for the next century | Yes | No | Don't use pesticides | Soil organisms have less harmful effects | Yes | No | No | Sterckeman T, Gossiaux L, Guimont S, Sirguey C, Lin Z. Sci Total Environ. 2018, 639:1440-1452. |
| 23 | Changes in soil oribatid communities associated with conversion from conventional to organic agriculture | Yes | No | Don't use pesticides | Improve soil quality | Yes | No | No | Khalil MA, Al-Assiuty AN, van Straalen NM, Al-Assiuty BA. Exp Appl Acarol. 2016, 68(2):183-96. |
| 24 | Coating of fertilizers by degradable polymers | No | Yes | Use pesticides | Adverse ecosystem | Yes | No | No | Devassine M, Henry F, Guerin P, Briand X. Int J Pharm. 2002, 242(1-2):399-404. |
| 25 | Commercial Organic Versus Conventional Whole Rye and Wheat Flours for Making Sourdough Bread: Safety, Nutritional, and Sensory Implications | Yes | No | Choose organic | Good quality | No | No | Yes | Pontonio E, et al. Front Microbiol. 2021, 12:674413. |
| 26 | Conservation Agriculture Improves Soil Quality, Crop Yield, and Incomes of Smallholder Farmers in North Western Ghana | Yes | No | Reduced energy consumption | Higher economic benefits | No | Yes | No | Naab JB, Mahama GY, Yahaya I, Prasad PVV. Front Plant Sci. 2017, 8:996. |
| 27 | Conservation agriculture improves yield and reduces weeding activity in sandy soils of Cambodia | Yes | No | Low input organic fertilizer artificial weeding and rice husk power generation | Reduced energy consumption | Yes | No | No | Edralin DA, Sigua GC, Reyes MR, Mulvaney MJ, Andrews SS. Agron Sustain. 2017, 37:52. |
| 28 | Consistent responses of the microbial community structure to organic farming along the middle and lower reaches of the Yangtze River | Yes | No | Don't use pesticides | Soil organisms have less harmful effects | Yes | No | No | Wang W, et al. Sci Rep. 2016, 6:35046. |
| 29 | CONTRASTING ARTHROPOD COMMUNITIES IN WOLFBERRY ORCHARDS OF DIFFERENT MANAGEMENT REGIMES IN NORTHWESTERN CHINA | Yes | No | Choose organic | Increased biodiversity | Yes | No | No | Liu J, Yan Y, Ali A, Han P, Ahmad F, Yu M. Pak. J. Agri. Sci. 2017, 54(3): 595-605. |
| 30 | Contrasting Patterns of Functional Diversity in Coffee Root Fungal Communities Associated with Organic and Conventionally Managed Fields | Yes | No | Don't use pesticides | Increased biodiversity | Yes | No | No | Sternhagen EC, et al. Appl Environ Microbiol. 2020, 86(11):e00052-20. |
| 31 | Contribution of organically grown crops to human health | No | Yes | Use pesticides | Adverse food safety | No | No | Yes | Johansson E, Hussain A, Kuktaite R, Andersson SC, Olsson ME. Int J Environ Res Public Health. 2014, 11(4):3870-93. |
| 32 | Cropping systems impact changes in soil fungal, but not prokaryote, alpha-diversity and community composition stability over a growing season in a long-term field trial | Yes | No | Don't use pesticides | Increased biodiversity | Yes | No | No | Finn DR, Lee S, Lanzén A, Bertrand M, Nicol GW, Hazard C. FEMS Microbiol Ecol. 2021, 97(10):fiab136. |
| 33 | Detection of bird nests during mechanical weeding by incremental background modeling and visual saliency | Yes | No | Weeding by machine | Increased biodiversity | Yes | No | No | Steen KA, Therkildsen OR, Green O, Karstoft H. Sensors (Basel). 2015, 15(3):5096-111. |
| 34 | Determination of pesticide residues in Turkey's table grapes: the effect of integrated pest management, organic farming, and conventional farming | Yes | No | Don't use pesticides | Ecosystem benefits | Yes | No | No | Turgut C, Ornek H, Cutright TJ. Environ Monit Assess. 2011, 173(1-4):315-23. |
| 35 | Distinct dynamics of Vibrio parahaemolyticus populations in two farming models | No | Yes | Use pesticides | Adverse food safety | No | No | Yes | Yang Q, et al. J Appl Microbiol. 2021. |
| 36 | Diversity and Abundance of Edaphic Arthropods Associated with Conventional and Organic Sugarcane Crops in Brazil | Yes | No | Don't use pesticides | Soil organisms have less harmful effects | Yes | No | No | dos Santos L A O, Naranjo-Guevara N, Fernandes O A. Florida Entomologist. 2017, 100(1): 134-144. |
| 37 | Does plant-Microbe interaction confer stress tolerance in plants: A review? | No | Yes | Use pesticides | Adverse ecosystem | Yes | No | No | Kumar A, Verma JP. Microbiol Res. 2018, 207:41-52. |
| 38 | Ecuadorian banana farms should consider organic banana with low price risks in their land-use portfolios | Yes | No | Consumer consciousness | Good marketing | No | Yes | No | Castro LM, Calvas B, Knoke T. PLoS One. 2015, 10(3):e0120384. |
| 39 | Effect of organic, conventional and mixed cultivation practices on soil microbial community structure and nematode abundance in a cultivated onion crop | Yes | No | Don't use pesticides | Increased biodiversity | Yes | No | No | Reilly K, et al. J Sci Food Agric. 2013, 93(15):3700-9. |
| 40 | Effects of agrochemicals on the beneficial plant rhizobacteria in agricultural systems | No | Yes | Use pesticides | Soil organisms have large deleterious effects | Yes | No | No | Aloo BN, Mbega ER, Makumba BA, Tumuhairwe JB. Environ Sci Pollut Res Int. 2021, 28(43):60406-60424. |
| 41 | Effects of Cultivation Systems and Environmental Conditions on Peppermint (Mentha x piperita L.) Biomass Yield and Oil Content | Yes | No | Don't use pesticides | Good quality | No | No | Yes | Oroian C, Covrig I, Odagiu A, MĂLINAȘ C, Moldovan C, FLEȘERIU A. Notulae Botanicae Horti Agrobotanici Cluj-Napoca. 2017, 45(2):576-581. |
| 42 | Effects of different cultivation patterns on soil aggregates and organic carbon fractions | Yes | No | Don't use pesticides | Improve soil quality | Yes | No | No | Qiu XL, Zong LG, Liu YF, Du XF, Luo M, Wang RC. Huan Jing Ke Xue. 2015, 36(3):1045-52. |
| 43 | Effects of organic and conventional crop management on vineyard biodiversity | Yes | No | Don't use pesticides | Increased biodiversity | Yes | No | No | Puig-Montserrat X, et al. Agriculture, ecosystems & environment, 2017, 243: 19-26. |
| 44 | Effects of simulated drought on biological soil quality, microbial diversity and yields under long-term conventional and organic agriculture | Yes | No | Don't use pesticides | Improve soil quality | Yes | No | No | Kundel D, et al. FEMS Microbiol Ecol. 2020, 96(12):fiaa205. |
| 45 | Energy efficiency, productivity and profitability of rice (Oryza sativa L.) based cropping systems for selected conservation practices | Yes | No | Reduced energy consumption | Higher economic benefits | No | Yes | No | Rautaray SK, Mishra A, Verma OP. Archives of Agronomy and Soil Science. 2017, 63(14): 1993-2006. |
| 46 | Energy input , output and economic analysis in organic production of guava ( Psidium guajava ) cv . Allahabad Safeda | Yes | No | Choose organic | Reduced energy consumption | Yes | No | No | Rautaray SK, Mishra A, Verma OP. Archives of Agronomy and Soil Science. 2017, 63(14):1993-2006. |
| 47 | Energy input in conventional and organic paddy rice production in Missouri and Italy: A comparative case study | Yes | No | Low input-output ratio | Higher economic benefits | No | Yes | No | Pagani M, Johnson TG, Vittuari M. J Environ Manage. 2017, 188:173-182. |
| 48 | Energy-environmental assessment of the UIA-OpenAgri case study as urban regeneration project through agriculture | Yes | No | Don't use pesticides | Reduced energy consumption | Yes | No | No | Caputo P, Zagarella F, Cusenza MA, Mistretta M, Cellura M. Sci Total Environ. 2020, 729:138819. |
| 49 | Environmental impacts and production performances of organic agriculture in China: A monetary valuation | No | Yes | Use pesticides | Adverse ecosystem | Yes | No | No | Meng F, Qiao Y, Wu W, Smith P, Scott S. J Environ Manage. 2017, 188:49-57. |
| 50 | Evaluating eco-friendly botanicals (natural plant extracts) as alternatives to synthetic fungicides | No | Yes | Use pesticides | Health | No | No | Yes | Al-Samarrai G, Singh H, Syarhabil M. Ann Agric Environ Med. 2012, 19(4):673-6. |
| 51 | Evaluation of the yield, nitrogen Use efficiency and adaptation in pakchoi cultivars under organic and conventional management systems during two successive seasons | Yes | No | Don't use pesticides | Good quality | No | No | Yes | Han R, Yang D, Hassani D, Wang X, Liu X, Huang D. Acta Agriculturae Scandinavica, Section B—Soil & Plant Science. 2017, 67(1):85-93. |
| 52 | Feeding partridges with organic or conventional grain triggers cascading effects in life-history traits | No | Yes | Use pesticides | Decreased biodiversity | Yes | No | No | Moreau J, et al. Environ Pollut. 2021 , 278:116851. |
| 53 | Fresh Compost Tea Application Does Not Change Rhizosphere Soil Bacterial Community Structure, and Has No Effects on Soybean Growth or Yield | No | Yes | Choose traditional | Decreased biodiversity | Yes | No | No | Bali R, Pineault J, Chagnon PL, Hijri M. Plants (Basel). 2021, 10(8):1638. |
| 54 | Functional diversity and dominant populations of bacteria in banana plantation soils as influenced by long-term organic and conventional farming | Yes | No | Don't use pesticides | Increased biodiversity | Yes | No | No | Chou YM, Shen FT, Chiang SC, Chang CM. Applied Soil Ecology. 2017, 110: 21-33. |
| 55 | Fungal communities are differentially affected by conventional and biodynamic agricultural management approaches in vineyard ecosystems | Yes | No | Don't use pesticides | Increased biodiversity | Yes | No | No | Morrison-Whittle P, Lee SA, Goddard MR. Agriculture, Ecosystems & Environment. 2017, 246:306-313. |
| 56 | Grazing of cover crops in integrated crop-livestock systems | No | Yes | Monoculture | Decreased biodiversity | Yes | No | No | Planisich A, Utsumi SA, Larripa M, Galli JR. Animal. 2021, 15(1):100054. |
| 57 | How and Why Does the Attitude-Behavior Gap Differ Between Product Categories of Sustainable Food? Analysis of Organic Food Purchases Based on Household Panel Data | Yes | No | Consumer consciousness | Good marketing | No | Yes | No | Schäufele I, Janssen M. Front Psychol. 2021, 12:595636. |
| 58 | Identification of two pesticide-tolerant bacteria isolated from Medicago sativa nodule useful for organic soil phytostabilization | Yes | No | Don't use pesticides | Improve soil quality | Yes | No | No | Aroua I, et al. Int Microbiol. 2019, 22(1):111-120. |
| 59 | Impact of 2,4-D and fipronil on the tropical midge Chironomus sancticaroli (Diptera: Chironomidae) | No | Yes | Use pesticides | Adverse ecosystem | Yes | No | No | Pinto TJDS, et al. Ecotoxicol Environ Saf. 2021, 209:111778. |
| 60 | Impact of seven years of organic farming on soil and produce quality and crop yields in eastern Himalayas, India | Yes | No | Choose organic | Soil fertility increases | Yes | No | No | Das A, et al. Agriculture, ecosystems & environment. 2017, 236:142-153. |
| 61 | Impacts of inorganic and organic fertilization treatments on bacterial and fungal communities in a paddy soil | Yes | No | Don't use pesticides | Increased biodiversity | Yes | No | No | Wang J, et al. Applied Soil Ecology. 2017, 112:42-50. |
| 62 | Influence of agricultural practices on fruit quality of bell pepper | Yes | No | Don't use pesticides | Good quality | No | No | Yes | Abu-Zahra TR. Pak J Biol Sci. 2011, 14(18):876-81. |
| 63 | Integrated modeling of extended agro-food supply chains: A systems approach | Yes | No | Choose organic | Ecosystem benefits | Yes | No | No | Taghikhah F, Voinov A, Shukla N, Filatova T, Anufriev M. Eur J Oper Res. 2021, 288(3):852-868. |
| 64 | Investigating dynamic interconnections between organic farming adoption and freshwater sustainability | Yes | No | Grower consciousness | Water | Yes | No | No | Aivazidou E, Tsolakis N. J Environ Manage. 2021, 294:112896. |
| 65 | Isolation, Characterization, and Evaluation of Native Rhizobacterial Consortia Developed From the Rhizosphere of Rice Grown in Organic State Sikkim, India, and Their Effect on Plant Growth | Yes | No | Cultivation of fungus-nourishing plants and use of organic fertilizers | Increased biodiversity | Yes | No | No | Sherpa MT, Sharma L, Bag N, Das S. Front Microbiol. 2021, 12:713660. |
| 66 | Landscape context affects the sustainability of organic farming systems | Yes | No | Don't use pesticides | Increased biodiversity | Yes | No | No | Smith OM, et al. Proc Natl Acad Sci U S A. 2020, 117(6):2870-2878. |
| 67 | Lettuce (Lactuca sativa) productivity influenced by microbial inocula under nitrogen-limited conditions in aquaponics | Yes | No | Choose organic | Ecosystem benefits | Yes | No | No | Day JA, et al. PLoS One. 2021, 16(2):e0247534. |
| 68 | Long-Term Chemical-Only Fertilization Induces a Diversity Decline and Deep Selection on the Soil Bacteria | No | Yes | Use pesticides | Decreased biodiversity | Yes | No | No | Xu Q, et al. mSystems. 2020, 5(4):e00337-20. |
| 69 | Long-term no-tillage and organic input management enhanced the diversity and stability of soil microbial community | Yes | No | No-tillage and ridge culture | Increased biodiversity | Yes | No | No | Wang Y, Li C, Tu C, Hoyt GD, DeForest JL, Hu S. Sci Total Environ. 2017, 609:341-347. |
| 70 | Metagenomic analysis reveals enhanced biodiversity and composting efficiency of lignocellulosic waste by thermoacidophilic effective microorganism (tEM) | Yes | No | Don't use pesticides | Increased biodiversity | Yes | No | No | B Henry A, Maung CEH, Kim KY Professor. J Environ Manage. 2020, 276:111252. |
| 71 | Metals and micronutrients in some edible crops and their cultivation soils in eastern-central region of Tunisia: A comparison between organic and conventional farming | No | Yes | Choose traditional | Adverse food safety | No | No | Yes | Hattab S, Bougattass I, Hassine R, Dridi-Al-Mohandes B. Food Chem. 2019, 270:293-298. |
| 72 | Mycotoxins and flours: Effect of type of crop, organic production, packaging type on the recovery of fungal genus and mycotoxins | No | Yes | Use pesticides | Adverse food safety | No | No | Yes | Sacco C, et al. Int J Food Microbiol. 2020, 334:108808. |
| 73 | Nanobiotechnological advancements in agriculture and food industry: Applications, nanotoxicity, and future perspectives | No | Yes | Use pesticides | Adverse food safety | No | No | Yes | Ali SS, et al. Sci Total Environ. 2021, 792:148359. |
|  |  | No | Yes | Use pesticides | Adverse ecosystem | Yes | No | No |  |
| 74 | Nanoparticles in sustainable agriculture: An emerging opportunity | No | Yes | Use pesticides | Adverse ecosystem | Yes | No | No | Singh RP, Handa R, Manchanda G. J Control Release. 2021, 1234-1248. |
| 75 | Nanovesicles from Organic Agriculture-Derived Fruits and Vegetables: Characterization and Functional Antioxidant Content | Yes | No | Choose organic | Good quality | No | No | Yes | Logozzi M, Di Raimo R, Mizzoni D, Fais S. Int J Mol Sci. 2021, 22(15):8170. |
| 76 | Nutritional quality of lettuce and onion as companion plants from organic and conventional production in north Greece | Yes | No | companion planting | Good quality | No | No | Yes | Kapoulas N, Koukounaras A, Ilić Z S. Scientia horticulturae. 2017, 219:310-318. |
| 77 | Organic agriculture promotes evenness and natural pest control | Yes | No | Promote balance between predators | Ecosystem benefits | Yes | No | No | Crowder DW, Northfield TD, Strand MR, Snyder WE. Nature. 2010, 466(7302):109-12. |
| 78 | Organic and conservation agriculture promote ecosystem multifunctionality | Yes | No | Choose organic | Ecosystem benefits | Yes | No | No | Wittwer RA, et al. Sci Adv. 2021, 7(34):eabg6995. |
| 79 | Organic farming increases richness of fungal taxa in the wheat phyllosphere | Yes | No | Don't use pesticides | Increased biodiversity | Yes | No | No | Karlsson I, Friberg H, Kolseth AK, Steinberg C, Persson P. Mol Ecol. 2017, 26(13):3424-3436. |
| 80 | Organic Farming Sharpens Plant Defenses in the Field | Yes | No | Don't use pesticides | Increased biodiversity | Yes | No | No | Krey KL, et al. Front Sustain Food Syst. 2020, 4:97. |
| 81 | Organic fertility inputs synergistically increase denitrification-derived nitrous oxide emissions in agroecosystems | Yes | No | Choose organic | Soil fertility increases | Yes | No | No | Saha D, Kaye JP, Bhowmik A, Bruns MA, Wallace JM, Kemanian AR. Ecol Appl. 2021, 31(7):e02403. |
| 82 | Organic foods: do they have a role? | Yes | No | Don't use pesticides | Ecosystem benefits | Yes | No | No | Köpke U. Forum Nutr. 2005, (57):62-72. |
| 83 | Paradise lost? Pesticide pollution in a European region with considerable amount of traditional agriculture | No | Yes | Use pesticides | Adverse ecosystem | Yes | No | No | Schreiner VC, et al. Water Res. 2021, 188:116528. |
| 84 | Performance of Winter Wheat Cultivars Grown Organically and Conventionally with Focus on Fusarium Head Blight and Fusarium Trichothecene Toxins | Yes | No | Don't use pesticides | Increased biodiversity | Yes | No | No | Góral T, Łukanowski A, Małuszyńska E, Stuper-Szablewska K, Buśko M, Perkowski J. Microorganisms. 2019, 7(10):439. |
| 85 | Phenolic antioxidant-linked anti-hyperglycemic properties of rye cultivars grown under conventional and organic production systems | Yes | No | Don't use pesticides | Improve product quality | No | No | Yes | Mishra LK, Sarkar D, Zwinger S, Shetty K. Journal of cereal science. 2017, 76:108-115. |
| 86 | Possibility to predict the yield of potatoes grown under two crop production systems on the basis of selected morphological and physiological plant indicators | No | Yes | Choose traditional | Good quality | No | No | Yes | Krystyna Z, Milena P. Plant, Soil and Environment. 2017, 63(4):165-170. |
| 87 | Procambarus clarkii as a bioindicator of heavy metal pollution sources in the lower Ebro River and Delta | No | Yes | Use pesticides | Adverse ecosystem | Yes | No | No | Suárez-Serrano A, Alcaraz C, Ibáñez C, Trobajo R, Barata C. Ecotoxicol Environ Saf. 2010, 73(3):280-6. |
| 88 | Reduced nitrate leaching and enhanced denitrifier activity and efficiency in organically fertilized soils | Yes | No | Don't use pesticides | Improve soil quality | Yes | No | No | Kramer SB, Reganold JP, Glover JD, Bohannan BJ, Mooney HA. Proc Natl Acad Sci U S A. 2006, 103(12):4522-7. |
| 89 | Rice-wheat cropping system: tillage, mulch, and nitrogen effects on soil carbon sequestration and crop productivity | Yes | No | No-tillage and ridge culture | Increased biodiversity | Yes | No | No | Adhikari KR, Dahal KR, Chen ZS, Tan YC, Lai JS. Paddy and water environment. 2017, 15(4):699-710. |
| 90 | Scenario modeling potential eco-efficiency gains from a transition to organic agriculture: life cycle perspectives on Canadian canola, corn, soy, and wheat production | Yes | No | Choose organic | Reduced energy consumption | Yes | No | No | Pelletier N, Arsenault N, Tyedmers P. Environ Manage. 2008, 42(6):989-1001. |
| 91 | Spider predation on rosy apple aphid in conventional, organic and insecticide-free orchards and its impact on aphid populations | Yes | No | Don't use pesticides | Increased biodiversity | Yes | No | No | Lefebvre M, Franck P, Olivares J, Ricard JM, Mandrin JF, Lavigne C. Biological Control. 2017, 104:57-65. |
| 92 | Subtle differences in birds detected between organic and nonorganic farms in Saskatchewan Prairie Parklands by farm pair and bird functional group | Yes | No | Don't use pesticides | Increased biodiversity | Yes | No | No | Kirk DA, Lindsay KEF. Agriculture, ecosystems & environment. 2017, 246:184-201. |
| 93 | The Association With Two Different Arbuscular Mycorrhizal Fungi Differently Affects Water Stress Tolerance in Tomato | Yes | No | Don't use pesticides | Soil organisms have less harmful effects | Yes | No | No | Volpe V, et al. Front Plant Sci. 2018, 9:1480. |
| 94 | THE COMPARATIVE ECONOMIC ANALYSIS OF ORGANIC AND CONVENTIONAL DRIED APRICOT PRODUCTION: A CASE STUDY FOR TURKEY | Yes | No | Perks | Higher economic benefits | No | Yes | No | Ucar K, Saner G, Engindeniz S. Fresenius Environmental Bulletin. 2017, 26(7):4555-4560. |
| 95 | The effect of earthworms on plant response in metal contaminated soil focusing on belowground-aboveground relationships | No | Yes | Use pesticides | Adverse ecosystem | Yes | No | No | Hullot O, Lamy I, Tiziani R, Mimmo T, Ciadamidaro L. Environ Pollut. 2021, 274:116499. |
| 96 | The impact of different weed management strategies on weed flora of wheat-based cropping systems | No | Yes | Use pesticides | Evolution of drug-resistant weeds | Yes | No | No | Shahzad M, et al. PLoS One. 2021, 16(2):e0247137. |
| 97 | THE INFLUENCE OF PLANT ACTIVATORS ON THE YIELD AND FRUIT QUALITY CHARACTERISTICS OF ORGANICALLY GROWN PEPPER | Yes | No | Don't use pesticides | Improve product quality | No | No | Yes | Unlu H, Unlu HO, Karakurt Y. FEB-FRESENIUS ENVIRONMENTAL BULLETIN. 2017:4305. |
| 98 | The use of bio-energy crops (Zea mays) for 'phytoattenuation' of heavy metals on moderately contaminated soils: a field experiment | No | Yes | Use pesticides | Adverse food safety | No | No | Yes | Meers E, et al. Chemosphere. 2010, 78(1):35-41. |
| 99 | Toxicity assessment of herbicides quizalafop-p-ethyl and clodinafop towards Rhizobium pea symbiosis | No | Yes | Use pesticides | Adverse ecosystem | Yes | No | No | Ahemad M, Khan MS. Bull Environ Contam Toxicol. 2009, 82(6):761-6. |
| 100 | Water table response to an experimental alley farming trial: dissecting the spatial and temporal structure of the data | No | Yes | Choose traditional | Soil fertility declines | Yes | No | No | Noorduijn SL, Ghadouani A, Vogwill R, Smettem KR, Legendre P. Ecol Appl. 2010, 20(6):1704-20. |
| 101 | Widespread Occurrence of Pesticides in Organically Managed Agricultural Soils-the Ghost of a Conventional Agricultural Past? | No | Yes | Use pesticides | Soil organisms have large deleterious effects | Yes | No | No | Riedo J, et al. Environ Sci Technol. 2021, 55(5):2919-2928. |
|  |  | No | Yes | Use pesticides | Decreased biodiversity | Yes | No | No |  |
| 102 | Work and technological innovation in organic agriculture | Yes | No | Consumer consciousness | Good marketing | No | Yes | No | Tereso MJ, et al. Work. 2012, 41 Suppl 1:4975-8. |
| 103 | Work exposures and mental and musculoskeletal symptoms in organic farming | Yes | No | Choose organic | Farmers' health | No | No | Yes | Mattila TEA, Perkiö-Mäkelä M, Hirvonen M, Kinnunen B, Väre M, Rautiainen RH. Ergonomics. 2021, 1-11. |
| 104 | Yield, quality and soil health under organic vs conventional farming in taro | Yes | No | Don't use pesticides | Improve soil quality | Yes | No | No | Suja G, Byju G, Jyothi AN, Veena SS, Sreekumar J. Scientia Horticulturae. 2017, 218:334-343. |
| 105 | Effects of rice-duck mutualistic organic farming on rice quality in the Yellow River Delta, China | Yes | No | Rice-duck cultivation | Ecosystem benefits | Yes | No | No | Wang JL, Li J, Cao YY. Ying Yong Sheng Tai Xue Bao. 2016, 27(7):2315-2320. |
| 106 | Mechanism and capacities of reducing ecological cost through rice-duck cultivation | Yes | No | Rice-duck cultivation | Higher economic benefits | No | Yes | No | Long P, et al. J Sci Food Agric. 2013, 93(12):2881-91. |
| 107 | Crop yeilds and economic comparisons of organic, low-input, and conventional farming systems in the California's Sacramento Valley | Yes | No | Low input-output ratio | Higher economic benefits | No | Yes | No | Clark S, Klonsky K, Livingston P, Temple S. American Journal of alternative agriculture. 1999, 14(3):109-121. |
| 108 | Generation of highly potent organic fertilizer from pernicious aquatic weed Salvinia molesta | Yes | No | Don't use pesticides | Increased biodiversity | Yes | No | No | Hussain N, Abbasi T, Abbasi SA. Environ Sci Pollut Res Int. 2018, 25(5):4989-5002. |
| 109 | In vivo assessment of plant extracts for control of plant diseases: A sesquiterpene ketolactone isolated from Curcuma zedoaria suppresses wheat leaf rust | Yes | No | Don't use pesticides | Ecosystem benefits | Yes | No | No | Han JW, et al. J Environ Sci Health B. 2018, 53(2):135-140. |
| 110 | Global changes in soil stocks of carbon, nitrogen, phosphorus, and sulphur as influenced by long-term agricultural production | Yes | No | Reduced energy consumption | Soil fertility increases | Yes | No | No | Kopittke PM, Dalal RC, Finn D, Menzies NW. Glob Chang Biol. 2017, 23(6):2509-2519. |
| 111 | Resource Legacies of Organic and Conventional Management Differentiate Soil Microbial Carbon Use | Yes | No | Don't use pesticides | Improve soil quality | Yes | No | No | Arcand MM, Levy-Booth DJ, Helgason BL. Front Microbiol. 2017, 8:2293. |
| 112 | A Diagnosis of Biophysical and Socio-Economic Factors Influencing Farmers' Choice to Adopt Organic or Conventional Farming Systems for Cotton Production | Yes | No | Perks | Higher economic benefits | No | Yes | No | Riar A, Mandloi LS, Poswal RS, Messmer MM, Bhullar GS. Front Plant Sci. 2017, 8:1289. |
| 113 | Higher Antioxidant Activity, Total Flavonols, and Specific Quercetin Glucosides in Two Different Onion (Allium cepa L.) Varieties Grown under Organic Production: Results from a 6-Year Field Study | Yes | No | Tillage method | Good quality | No | No | Yes | Ren F, Reilly K, Kerry JP, Gaffney M, Hossain M, Rai DK. J Agric Food Chem. 2017, 65(25):5122-5132. |
| 114 | The footprint of marginal agriculture in the Mediterranean mountain landscape: An analysis of the Central Spanish Pyrenees | No | Yes | Use pesticides | Adverse ecosystem | Yes | No | No | Lasanta T, Nadal-Romero E, Errea MP. Sci Total Environ. 2017, 599-600:1823-1836. |
| 115 | Characterization factors for land use impacts on biodiversity in life cycle assessment based on direct measures of plant species richness in European farmland in the 'Temperate Broadleaf and Mixed Forest' biome | Yes | No | Don't use pesticides | Increased biodiversity | Yes | No | No | Knudsen MT, et al. Sci Total Environ. 2017, 580:358-366. |
| 116 | Impact of Cropping Systems, Soil Inoculum, and Plant Species Identity on Soil Bacterial Community Structure | Yes | No | Don't use pesticides | Soil organisms have less harmful effects | Yes | No | No | Ishaq SL, et al. Microb Ecol. 2017, 73(2):417-434. |
| 117 | Sensitivity of the farmland bird community to crop diversification in Sweden: does the CAP fit? | Yes | No | Don't use pesticides | Increased biodiversity | Yes | No | No | Josefsson J, Berg Å, Hiron M, Pärt T, Eggers S. Journal of Applied Ecology. 2017, 54(2):518-526. |
| 118 | Diversity of arbuscular mycorrhizal fungi in Brazil’s Caatinga and experimental agroecosystems | Yes | No | Don't use pesticides | Soil organisms have less harmful effects | Yes | No | No | Pontes JS, et al. Biotropica. 2017, 49(3):413-427. |
| 119 | Species diversity and community composition of native arbuscular mycorrhizal fungi in apple roots are affected by site and orchard management | Yes | No | Don't use pesticides | Increased biodiversity | Yes | No | No | Turrini A, et al. Applied Soil Ecology. 2017, 116:42-54. |
| 120 | Does organic farming enhance biodiversity in Mediterranean vineyards? A case study with bats and arachnids | Yes | No | Don't use pesticides | Increased biodiversity | Yes | No | No | Froidevaux JSP, Louboutin B, Jones G. Agric Ecosyst Environ. 2017, 249:112-122. |
| 121 | Management matters: A comparison of ant assemblages in organic and conventional vineyards | Yes | No | Don't use pesticides | Increased biodiversity | Yes | No | No | Masoni A, et al. Agriculture, Ecosystems & Environment. 2017, 246:175-183. |
| 122 | Soil Microbiome Is More Heterogeneous in Organic Than in Conventional Farming System | No | Yes | Use pesticides | Decreased biodiversity | Yes | No | No | Lupatini M, Korthals GW, de Hollander M, Janssens TK, Kuramae EE. Front Microbiol. 2017, 7:2064. |
| 123 | Why farming with high tech methods should integrate elements of organic agriculture | Yes | No | Reduced energy consumption | Soil fertility increases | Yes | No | No | Ammann K. N Biotechnol. 2009, 25(6):378-88. |
| 124 | Crop yield and energy use in organic and conventional farming: A case study in north-east Italy | Yes | No | Reduced energy consumption | Soil fertility increases | Yes | No | No | Dal Ferro N, Zanin G, Borin M. European Journal of Agronomy. 2017, 86: 37-47. |
| 125 | Changes in soil mesofauna structure due to different land use systems in south Minas Gerais, Brazil | No | Yes | Use pesticides | Adverse ecosystem | Yes | No | No | Menezes-Oliveira VB, Bianchi MO, Espíndola ELG. Environ Monit Assess. 2021, 193(7):431. |
| 126 | Effect of Low-Input Organic and Conventional Farming Systems on Maize Rhizosphere in Two Portuguese Open-Pollinated Varieties (OPV), "Pigarro" (Improved Landrace) and "SinPre" (a Composite Cross Population) | No | Yes | Choose traditional | Adverse ecosystem | Yes | No | No | Ares A, et al. Front Microbiol. 2021, 26;12:636009. |
| 127 | Colonization of Aspergillus carbonarius and accumulation of ochratoxin A in Vitis vinifera, Vitis labrusca, and hybrid grapes - research on the most promising alternatives for organic viticulture | Yes | No | Choose traditional | Ecosystem benefits | Yes | No | No | Veras FF, Dachery B, Manfroi V, Welke JE. J Sci Food Agric. 2021, 101(6):2414-2421. |
| 128 | Biologia Futura: landscape perspectives on farmland biodiversity conservation | No | Yes | Choose organic | Adverse ecosystem | Yes | No | No | Batáry P, Báldi A, Ekroos J, Gallé R, Grass I, Tscharntke T. Biol Futur. 2020, 71(1-2):9-18. |

# Supplementary Table 2 Socio-demographic profiles of the respondents

| **Variable** | **N** | **%** |
| --- | --- | --- |
| **Gender** |  |  |
| Male | 432 | 42.94 |
| Female | 574 | 57.06 |
| **Age** |  |  |
| Below 18 | 7 | 0.70 |
| 18–24 | 147 | 14.61 |
| 25–29 | 174 | 17.29 |
| 30–39 | 219 | 21.77 |
| 40-50 | 324 | 32.21 |
| More than 50 | 135 | 13.42 |
| **Education** |  |  |
| Middle school or less | 49 | 4.87 |
| High school | 59 | 5.86 |
| Junior college degree | 199 | 19.78 |
| Bachelor degree | 457 | 45.43 |
| Master or PhD | 242 | 24.06 |
| **Income/year** |  |  |
| 50,000 RMB or less | 196 | 19.48 |
| 50000－100,000 RMB | 381 | 37.87 |
| 100,001－200,000 RMB | 327 | 32.51 |
| 200,001－500,000 RMB | 89 | 8.85 |
| More than 500,000 RMB | 13 | 1.29 |

**Supplementary Table 3** The global organic agricultural land and organic share in the agricultural from 1999 to 2019

| Year | Organic share | Organic agricultural land |
| --- | --- | --- |
| 1999 | 0.30 | 10.00 |
| 2000 | 0.30 | 11.00 |
| 2001 | 0.40 | 15.00 |
| 2002 | 0.50 | 17.30 |
| 2003 | 0.60 | 19.90 |
| 2004 | 0.60 | 25.80 |
| 2005 | 0.60 | 30.00 |
| 2006 | 0.70 | 29.20 |
| 2007 | 0.70 | 30.20 |
| 2008 | 0.80 | 31.50 |
| 2009 | 0.80 | 34.50 |
| 2010 | 0.80 | 36.30 |
| 2011 | 0.80 | 35.70 |
| 2012 | 0.90 | 36.70 |
| 2013 | 1.00 | 36.80 |
| 2014 | 1.00 | 43.10 |
| 2015 | 1.20 | 48.70 |
| 2016 | 1.40 | 50.40 |
| 2017 | 1.40 | 58.10 |
| 2018 | 1.50 | 69.40 |
| 2019 | 1.50 | 71.20 |

**Supplementary Table 4** The driving factors in the organic area share

| Countries | Organic shares (%) | GDP (dollar) | GDP per capita (dollar) | Arable land (%) | Agricultural added value in total GDP (%) | Industrial added value to GDP(%) |
| --- | --- | --- | --- | --- | --- | --- |
| United States | 0.0060 | 21420000000000.00 | 65279.50 | 0.1724 | 0.0092 | 0.1816 |
| China | 0.0040 | 14280000000000.00 | 10216.60 | 0.1268 | 0.0714 | 0.3859 |
| Germany | 0.0970 | 3860000000000.00 | 46467.50 | 0.3358 | 0.0072 | 0.2670 |
| India | 0.0130 | 2870000000000.00 | 2100.80 | 0.5261 | 0.1668 | 0.2418 |
| United Kingdom | 0.0260 | 2830000000000.00 | 42354.40 | 0.2495 | 0.0059 | 0.1783 |
| France | 0.1130 | 2710000000000.00 | 40380.10 | 0.3310 | 0.0159 | 0.1713 |
| Italy | 0.1520 | 200000000000.00 | 33566.80 | 0.2258 | 0.0191 | 0.2138 |
| Brazil | 0.0050 | 1880000000000.00 | 8897.50 | 0.0667 | 0.0440 | 0.1840 |
| Canada | 0.0230 | 1740000000000.00 | 46326.70 | 0.0431 | - | - |
| Austria | 0.2610 | 445000000000.00 | 50121.50 | 0.1608 | 0.0106 | 0.2552 |
| Switzerland | 0.1650 | 731000000000.00 | 85300.30 | 0.1007 | 0.0066 | 0.2483 |
| Uruguay | 0.1530 | 61231149881.00 | 17688.00 | 0.1129 | 0.0645 | 0.1785 |
| Australia | 0.0990 | 1396570000000.00 | 55057.20 | 0.0402 | 0.0212 | 0.2538 |
| Liechtenstein | 0.4100 | 6839145106.86 | 180366.72 | 0.1350 | 0.1376 | 0.4086 |
| Sao Tomé and Principe | 0.2490 | 427425039.68 | 1987.58 | 0.0417 | 0.1229 | 0.1344 |
| Estonia | 0.2230 | 31471100656.24 | 23717.80 | 0.1583 | 0.0249 | 0.2204 |
| Sweden | 0.2040 | 531283304459.67 | 51686.85 | 0.0626 | 0.0138 | 0.2221 |
| Czech Republic | 0.1540 | 250686478649.06 | 23490.40 | 0.3218 | 0.0194 | 0.3146 |
| latvia | 0.1480 | 34055464655.33 | 17794.48 | 0.2086 | 0.0023 | 0.1858 |
| Samoa | 0.1450 | 852250190.99 | 4324.10 | 0.1148 | 0.0975 | 0.1559 |
| Finland | 0.1350 | 268966065200.00 | 48711.56 | 0.0738 | 0.0245 | 0.2393 |
| Denmark | 0.1090 | 350104327658.68 | 60213.09 | 0.5980 | 0.0133 | 0.2112 |
| Slovenia | 0.1030 | 54174227308.89 | 25940.73 | 0.0903 | 0.0201 | 0.2889 |
| Slovakia | 0.1030 | 105119160234.12 | 19273.25 | 0.2804 | 0.0248 | 0.2838 |
| Spain | 0.0970 | 1393490524517.64 | 29564.74 | 0.2379 | 0.0261 | 0.2048 |
| Greece | 0.0870 | 205326724570.23 | 19150.79 | 0.1658 | 0.0378 | 0.1289 |
| Timor-Leste | 0.0850 | 2017924900.00 | 1560.51 | 0.1042 | 0.1419 | 0.2937 |
| Faroe Islands | 0.0840 | 3126293219.78 | 64225.26 | 0.0215 | 0.1817 | 0.1855 |
| Portugal | 0.0820 | 239510770948.27 | 23284.53 | 0.1003 | 0.0205 | 0.1886 |
| Lithuania | 0.0810 | 54639938780.24 | 19555.21 | 0.3377 | 0.0322 | 0.2525 |
| Netherlands | 0.0370 | 907050863145.10 | 52295.04 | 0.3032 | 0.0165 | 0.1770 |
| turkey | 0.0140 | 761428183369.17 | 9126.59 | 0.2563 | 0.0642 | 0.2719 |
| Mexico | 0.0030 | 1268870527160.03 | 9946.03 | 0.1230 | 0.0339 | 0.3088 |
| Poland | 0.0350 | 595862086928.21 | 15694.84 | 0.3596 | 0.0235 | 0.2798 |
| Cyprus | 0.0500 | 24949065263.63 | 28288.46 | 0.1126 | 0.0174 | 0.1271 |
| Croatia | 0.0770 | 60752588976.32 | 14944.36 | 0.1421 | 0.0292 | 0.2026 |
| Belgium | 0.0720 | 533254518108.23 | 46414.44 | 0.2807 | 0.0063 | 0.1911 |
| Hungary | 0.0570 | 163503650313.08 | 1272.49 | 0.4738 | 0.0332 | 0.2497 |
| Dominican Republic | 0.0550 | 88941299733.50 | 8282.12 | 0.1815 | 0.0523 | 0.2908 |
| Fiji | 0.0530 | 5496250694.32 | 6175.87 | 0.0903 | 0.1185 | 0.1559 |

**Supplementary Table 5** Judgment matrix and its consistency test

| A1 | | | | | | | | |
| --- | --- | --- | --- | --- | --- | --- | --- | --- |
|  | E1 | E2 | E3 | E4 | Weight | | Consistency test | |
| E1 | 1 | 1/3 | 2 | 5 | 0.2370 | | λ_max_=4.002886898  CR=0.009623 | |
| E2 | 3 | 1 | 5 | 7 | 0.5632 | |  |  |
| E3 | 1/2 | 1/5 | 1 | 4 | 0.1456 | |  |  |
| E4 | 1/5 | 1/7 | 1/4 | 1 | 0.0542 | |  |  |
| A2 | | | | | | | | |
|  | E5 | E6 | E7  4  3  1 | | | Weight | | Consistency test |
| E5 | 1 | 2 |  |  |  | 0.5772 | | λ_max_=3.018324794  CR=0.070480 |
| E6 | 1/2 | 1 |  |  |  | 0.2952 | |  |
| E7 | 1/4 | 1/3 |  |  |  | 0.1276 | |  |

**Supplementary Table 6** The result of the input combined weight

|  | E1 | E2 | E3 | E4 |  |
| --- | --- | --- | --- | --- | --- |
| Organic agriculture | 0.25 | 0.75 | 0.6667 | 0.1667 | 1.833400 |
| Traditional agriculture | 0.75 | 0.25 | 0.3333 | 0.8333 | 2.166600 |

**Supplementary Table 7** The result of the income combined weight

|  | E5 | E6 | E7 |  |
| --- | --- | --- | --- | --- |
| Organic agriculture | 0.3333 | 0.6667 | 0.6667 | 1.6667 |
| Traditional agriculture | 0.6667 | 0.3333 | 0.3333 | 1.3333 |

(E1: Agrochemicals; E2: Labor; E3: [O](../../../../C:/Users/dell/AppData/Local/youdao/dict/Application/8.9.6.0/resultui/html/index.html#/javascript:;)rganic [fertilizer](../../../../C:/Users/dell/AppData/Local/youdao/dict/Application/8.9.6.0/resultui/html/index.html#/javascript:;); E4: Field management;

E5: Yield; E6: Quality; E7: Retail price).

**Supplementary Table 8** The profit comparison between ecological agriculture and traditional agriculture

| Mode | Input costs/667m^2^ | Yield/667m^2^ | [R](../../../../C:/Users/dell/AppData/Local/youdao/dict/Application/8.9.6.0/resultui/html/index.html#/javascript:;)etail [price](../../../../C:/Users/dell/AppData/Local/youdao/dict/Application/8.9.6.0/resultui/html/index.html#/javascript:;)/kg | Output value/667m^2^ | Income/667m^2^ | Input-output ratio |
| --- | --- | --- | --- | --- | --- | --- |
| Organic agriculture | 4100 | 120kg | 130 | 15600 | 11500 | 1:3.8 |
| Traditional agriculture | 2400 | 150kg | 40 | 6000 | 3600 | 1:2.5 |

**Supplementary Table 9** Odds ratios with fixed and random effects of selected covariates for the willingness obtained from two-level random intercept logistic regression model

| Variables | Estimate | Odds ratio | *p*-value |
| --- | --- | --- | --- |
| Age | 0307 | 1.359 | 0.042* |
| Education | 0.523 | 1.688 | 0.003** |
| Income/year | 0.742 | 2.099 | 0.005** |
| Health risk attitudes | -1.279 | 0.278 | 0.000** |
| Cognition and attitude | 2.733 | 0.472 | 0.033* |
| Retail price | -0.750 | 15.378 | 0.000** |
| Constant | -1.245 | 0.288 | 0.231 |

*: *P* < 0.05 **: *P* < 0.01

**Supplementary Table 10** The active components of *S. divaricata* in the imitation wild and cultivated

| No. | mode | prim-*O*-glucosylcimifugin(μg/g) | cimifugin  (μg/g) | 1. *O*-methylvisammioside   (μg/g) | sec-*O*-glucosylhamaudol (μg/g) |
| --- | --- | --- | --- | --- | --- |
| 1 | Organic cultivation | 9.1304 | 0.8639 | 2.9957 | 0.5403 |
| 2 | Conventional cultivation | 4.5435 | 0.2306 | 6.5381 | 0.3278 |
| 3 | Organic cultivation | 15.8656 | 0.3592 | 11.9860 | 0.6637 |
| 4 | Conventional cultivation | 6.9321 | 0.2949 | 0.5923 | 0.5923 |

Supplementary Table 11 Impact indicators adopted in the comparison of organic and conventional agriculture of medicinal plants

| Impact category | Impact indicator | Rationales | Conventional  Farming/ha | Price/RMB | Economic value in organic agriculture /RMB |
| --- | --- | --- | --- | --- | --- |
| **Economic** | Yield | Key function of agriculture | 3000kg  (*A. mongholicus*) | 20/kg | 124×10^6^ ↓ |
|  |  |  | 12000kg  (*P. lactiflora*) | 45/kg |  |
|  |  |  | 1500kg  (*C. deserticola* ) | 150/kg |  |
| **Environmental impacts** | Purchased fertilizer | Synthetic fertilizer was purchased in conventional agriculture. | 150kg | 2000/t | 4×10^6^ ↓ |
|  | Purchased pesticide and other pest control materials | In organic, pest control materials accounted for a small proportion of the variable costs, so were not considered. Chemical pesticide was purchased in convention. | 1.5kg | 300/kg | 2.7×10^6^ ↓ |
|  | Nitrogen leaching | Less nitrate leaching in organic due to no synthetic fertilizer inputs. | 10/kg | 100/kg | 6×10^6^ ↓ |
|  | Biodiversity | Beneficial effects on fauna and flora, landscape and ecosystem functions due to no synthetic fertilizer and pesticide applied and the use of environmental friendly farming measures (e.g., rotation). | Lack of appropriate methodologies due to the special nature of medicinal plants. | | |
| **Social well being** | Social well-being | The reduced use of pesticides reduces the risk of pesticide poisoning | 5000/person | 2 person/year | 10000 ↓ |

**Supplementary Table 12** The results of the correlation analysis between GDP and the share of organic agriculture

|  | | | GDP | The share of organic agriculture |
| --- | --- | --- | --- | --- |
| Spearman's correlation coefficient | GDP | *r* | 1 | -0.403* |
|  |  | significance | － | 0.011 |
|  | The share of organic agriculture | *r* | -0.403* | 1.000 |
|  |  | significance | 0.011 | － |

**Supplementary Table 13** Odds ratios with fixed and random effects of selected covariates for the farmers willingness obtained from two-level random intercept logistic regression model.

| Variables | Estimate | Odds ratio | *p*-value |
| --- | --- | --- | --- |
| Biodiversity | 0.397 | 1.488 | 0.002** |
| Input/output ratio | -6.522 | 0.001 | 0.017* |
| Pesticide poisoning | -1.854 | 0.157 | 0.020* |
| Constant | -4.490 | 0.011 | 0.124 |

*: *p* < 0.05 **: *p* < 0.01
